# Optimization of rPDT fusion protein expression by *Escherichia coli* in pilot scale fermentation: a statistical experimental design approach

**DOI:** 10.1186/s13568-018-0667-3

**Published:** 2018-08-22

**Authors:** Nasser Nassiri Koopaei, Parissa Khadiv-Parsi, Mohammad Reza Khoshayand, Mohammad Ali Mazlomi, Abbas Kebriaeezadeh, Hamid Moloudian, Roya Solhi, Mahdi Aminian

**Affiliations:** 1 Department of Pharmaceutical Engineering, School of Chemical Engineering, University of Tehran, Tehran, Iran; 2 Department of Clinical Biochemistry, Faculty of Medicine, Tehran University of Medical Sciences, Tehran, Iran; 3 Department of Food Control and Analysis, Faculty of Pharmacy, Tehran University of Medical Sciences, Tehran, Iran; 4 Department of Medical Biotechnology, School of Advanced Technologies in Medicine, Tehran University of Medical Sciences, Tehran, Iran; 5 Department of Pharmacoeconomics and Pharmaceutical Administration, Faculty of Pharmacy, Tehran University of Medical Sciences, Tehran, Iran; 6 Recombinant Vaccine Research Center, Tehran University of Medical Sciences, Tehran, Iran

**Keywords:** Bacterial toxin, Box–Behnken design, Fermentation optimization, Recombinant fusion protein, Statistical experimental design

## Abstract

High yield recombinant protein production is highly desirable for biotechnological purposes. In the design of recombinant expression conditions, a number of essential central elements such as expression strain, type of medium, bioprocess optimization, and mathematical modeling should be considered. Well-designed industrial scale production of one recombinant protein with optimized influential parameters and yield can address the cost and production reproducibility issues. In the present study, statistical experimental design methodology was used to investigate the effect of fermentation conditions (dissolved oxygen, IPTG, and temperature) on rPDT production by *Escherichia coli.* rPDT is a recombinant fusion protein consisting of three different protein domains including the N-terminal 179 amino acid fragment of the S1 subunit of pertussis toxin, the full-length genetically detoxified diphtheria toxin (CRM197), and the 50 kDa tetanus toxin fragment C. A 15 Box–Behnken design augmented with center points revealed that IPTG and DO at the center point and low temperature will result in high yield. The optimal condition for rPDT production were found to be 100 µM IPTG, DO 30% and temperature 20 °C.

## Introduction

Recombinant DNA technology has made it possible to produce different recombinant proteins in amounts required for research, clinical and industrial purposes (Rosano and Ceccarelli 2014; Pavlou and Reichert 2004; Kebriaeezadeh et al. 2013). *Escherichia coli* is known as a preferable expression system, because of its rapid growth and simplicity of cultivation. In spite of all the advantages, production of recombinant proteins involves very complicated steps that require application of sophisticated control and optimization approach (Andersen and Krummen 2002). Therefore, the critical task is to gain a great wealth of knowledge about the variables and responses related to the process yield and quality attributes of the product (Assenberg et al. 2013; Papaneophytou and Kontopidis 2014).

Design of experiments (DoE) defined as the statistical techniques used for planning, conducting, analyzing and interpretation of experimental data, provides powerful means to manage process parameters to optimize the results (Marini et al. 2014). Replacing the One-Variable-at-a-Time (OVAT) approach in design of experiments, DoE makes it possible to simultaneously investigate different parameters affecting the response (Papaneophytou and Kontopidis 2014). Regarding the wide range of influential factors in protein expression process, a screening survey would be necessary as a first step. Thereafter, optimization step should be done to find the optimal production process in which the selected design space includes plenty of variables. However, the most important interactions should be selected and introduced into the final model (Ellis 2001; Khoshayand et al. 2011; Hussain et al. 2008; Bayat et al. 2015).

In the present study, the bacterial strain expressing rPDT protein was selected and the rPDT production was optimized using statistical design. rPDT protein composes of the immunoprotective S1 fragment of pertussis toxin, the full-length nontoxic diphtheria toxin (CRM197), and fragment C of tetanus toxin (Aminian et al. 2007; Eisel et al. 1986). This fusion protein has been expressed in *E. coli* carrying the recombinant plasmid (pCoPDT) and has a molecular weight of 161 kDa that is recognizable by specific antibodies against the three toxins. rPDT expression is inducible by IPTG (Aminian et al. 2007; Esposito and Chatterjee 2006; Soria-Guerra et al. 2009). rPDT is a fusion protein containing the immunoprotective S1 fragment of pertussis toxin, the full-length non-toxic diphtheria toxin and fragment C of tetanus toxin which has been engineered to serve as a candidate for the vaccination against diphtheria–tetanus–pertussis (Aminian et al. 2007). However, the satisfactory protein yield challenge should be addressed. rPDT is a fusion protein and vaccine candidate which is not expressed in high amounts in bacteria. This paper tries to optimize rPDT production using DOE and develop model-based scalable process for similar protein production platforms particularly for fusion proteins. Considering the importance of recombinant fusion protein production using DOE, the present study intended to find the optimized condition for rPDT protein in a 5-l fermenter using the Box–Behnken statistical design. The aim was to produce adequate amount of rPDT fusion protein in a scalable and reproducible process to be able to run biochemical and immunological challenge tests and develop rPDT as a vaccine candidate through human clinical trials.

## Materials and methods

### Chemicals and media

Solvents and chemical reagents were analytical grade from Sigma-Aldrich. All the medium ingredients were obtained from Merck, Germany. LB (Luria–Bertani) medium is composed of NaCl 1% (W/V), Peptone 1% (W/V) and yeast 0.5% (W/V).

### Instruments

UV–visible spectrophotometer (PerkinElmer, Lambda 25) was used for OD determination. New Brunswick fermenter (model Bioflo 310, New Brunswick Scientific, Edison, NJ, USA) with 5 l working volume vessel was used for the fermentation runs. Sodium dodecyl sulfate polyacrylamide gel electrophoresis (SDS-PAGE) analysis and Blotting were performed using BioRad electrophoresis systems.

### Strain and plasmid

*Escherichia coli* Rosetta-gami2 carrying the recombinant plasmid (pET28a-pdt) was used for rPDT expression. The pET28a-pdt plasmid which was constructed in previous study is resistant to kanamycin (R) (Aminian et al. 2007). The rPDT protein production was inducible with IPTG. The bacteria were maintained in − 80 °C freezer in 50% glycerol.

### Fermentation conditions

The optimization studies were conducted in 5 l fermenter (New Brunswick BioFlo 310, NJ, USA) containing 3 l LB medium. All cultures contained 50 mg/l kanamycin. The bacteria were revived from frozen glycerol stocks following overnight culture in 2 ml LB containing 50 µg/ml kanamycin. To prepare the inoculum, the bacteria were added to a flask containing 150 ml LB broth, kanamycin 50 mg/l and incubated until the OD reached to 1 at 37 °C under shaking speed of 180 rpm (IKA, KS 4000). Then, 150 ml of culture was inoculated into the fermenter and the fermentation was operated at 37 °C. The pH was controlled at 7.2 using 10% NaOH and 10% H_2_SO_4_. The dissolved oxygen (DO) was maintained at 30% while agitation and air flow rate were cascaded with no pure oxygen purge. Primarily, the bacteria carrying the desired plasmid was cultured for 6 h withdrawing 2 ml samples every 1 h and measuring the turbidity at 600 nm to study the cell growth pattern. We used the results of this step to account for the growth phase design prior to the induction phase. The primary growth study showed that the culture should grow for about 4 h to achieve an optical density of 1.5 at 600 nm. Then, IPTG was added to the fermentation culture to induce the expression of rPDT protein. The expression phase of fermentation was operated at different operational conditions generated via statistical design for optimization studies. The fermentation runs terminated after further 6 h cultivation, the cultures were centrifuged at 5000*g* for 5 min in portions and 2 ml samples were taken for further studies.

### Western blot analysis

Protein expression was assessed by Western blotting. At the end of each expression run of 6 h, 2 ml samples of fermentation were centrifuged (5000*g* for 10 min at 4 °C) and subsequently the pellet was resuspended in a mixture of 100 µl of 50 mM phosphate buffer (pH 7.0), 2-mercaptoethanol (5%), 10% SDS and 0.004% bromophenol blue heated to boil for 5 min. The proteins were resolved by SDS-PAGE and transferred to nitrocellulose membranes according to the method of Towbin et al. (1979). Non-specific binding sites were blocked by submerging the membrane in 3% skim milk in TBST (0.1% Tween in TBS) for 1.5 h. The membrane was then incubated with anti-His tag monoclonal antibody (1/7500, Sigma-Aldrich) which was followed by incubation with the peroxidase-conjugated goat anti-mouse IgG antibody (1/1000, Sigma-Aldrich). Extensive washing was carried out after each step. rPDT protein was detected by adding of the horseradish peroxidase (HRP) substrate (7.5 mg 3,3′ diaminobenzidine (DAB), 10 µl 30% H_2_O_2_ in 15 ml TBST). Then, ImageJ software was employed to analyze the protein bands on the nitrocellulose papers.

### Experimental design

In a preliminary study, Plackett–Burman (PB) design was employed to screen factors affecting rPDT expression. This design can be used to select the most important factors among many candidates in order to make the study smaller and more manageable. Hence, using a PB factorial design, each factor was examined in two coded levels: − 1 and + 1 for low and high level, respectively. A first-order multiple regression was used to model the data, when no interaction between the main factors is assumed. Using the statistical software package Design-Expert software version 7.0.0 (Stat-Ease, Inc., Minneapolis, MN, USA), the design matrix was built for the evaluation of 8 variables in 12 experiments. All experiments were carried out in triplicate and the average of the levels of rPDT (band intensity) was taken as response. Different factors were screened for their effects on the rPDT expression (In Publishing). After recognition of the most influential parameters affecting rPDT production by *E. coli* from the previous study, Box–Behnken response surface methodology approach was applied to determine the optimum levels of these variables towards the protein production in the fermenter with the scale-up approach. Temperature, IPTG concentration, and DO were selected as factors for the optimization of rPDT expression each at three different levels coded as − 1, 0, and 1. The coded and actual values of the parameters are presented in Table 1. Table 2 shows the Box–Behnken design matrix generated by Design-Expert software version 7.0.0 (Stat-Ease, Inc., Minneapolis, MN, USA) with a total number of 15 experiments including 12 factorial points and 3 center point replications. rPDT expression was assessed as the response. Predicted rPDT expression was calculated using the following quadratic polynomial equation:

1 $${\text{Y}} = \, \beta_{0} + \, \beta_{ 1} {\text{X}}_{ 1} + \, \beta_{ 2} {\text{X}}_{ 2} + \, \beta_{ 3} {\text{X}}_{ 3} + \, \beta_{ 1 2} {\text{X}}_{ 1} {\text{X}}_{ 2} + \, \beta_{ 1 3} {\text{X}}_{ 1} {\text{X}}_{ 3} + \, \beta_{ 2 3} {\text{X}}_{ 2} {\text{X}}_{ 3} + \, \beta_{ 1 1} {\text{X}}_{ 1}^{ 2} + \, \beta_{ 2 2} {\text{X}}_{ 2}^{ 2} + \, \beta_{ 3 3} {\text{X}}_{ 3}^{ 2}$$

in which Y is the predicted response, β_0_ is the intercept, β_s_ are linear coefficients, β_ss_ are squared coefficients, X_sz_ are interaction coefficients, and X_1_, X_2_ and X_3_ are independent variables.

**Table 1:** Coded and real values for the Box–Behnken design

Variable	Variable code	− 1	0	+ 1
Temperature (°C)	X_1_	20	30	40
DO (%)	X_2_	10	30	50
IPTG (μM)	X_3_	25	112.5	200

**Table 2:** Box–Behnken design matrix

Run	T	DO	IPTG	rPDT band intensity
1	20	10	112.5	10934.5
2	40	10	112.5	6874.91
3	20	50	112.5	11531.7
4	40	50	112.5	6147.456
5	20	30	25	10745.06
6	40	30	25	5949.517
7	20	30	200	11619.7
8	40	30	200	6395.547
9	30	10	25	8968.991
10	30	50	25	9389.53
11	30	10	200	9104.195
12	30	50	200	9437.547
13	30	30	112.5	9953.16
14	30	30	112.5	10302.3
15	30	30	112.5	10235

Assuming this equation, the linear, quadratic and interactions of the independent variables on the response could be evaluated. Design-Expert software package was used to perform the statistical analysis and generate the graph plots.

The effect of independent variables on the response was assessed using ANOVA and a p-value of < 0.05 was used as the results significance level. Multiple correlation coefficient (R^2^) and adjusted R^2^ were used as quality indicators to assess the quadratic polynomial equation fitness. Relationship and interactions between the variables and the response was illustrated through contour and three-dimensional surface plots. Solving the second order equation from the model or grid search in RSM plots produced the optimal points.

## Results

### Growth kinetic and rPDT production by *E. coli* at basal fermentation condition

**Fig. 1:**
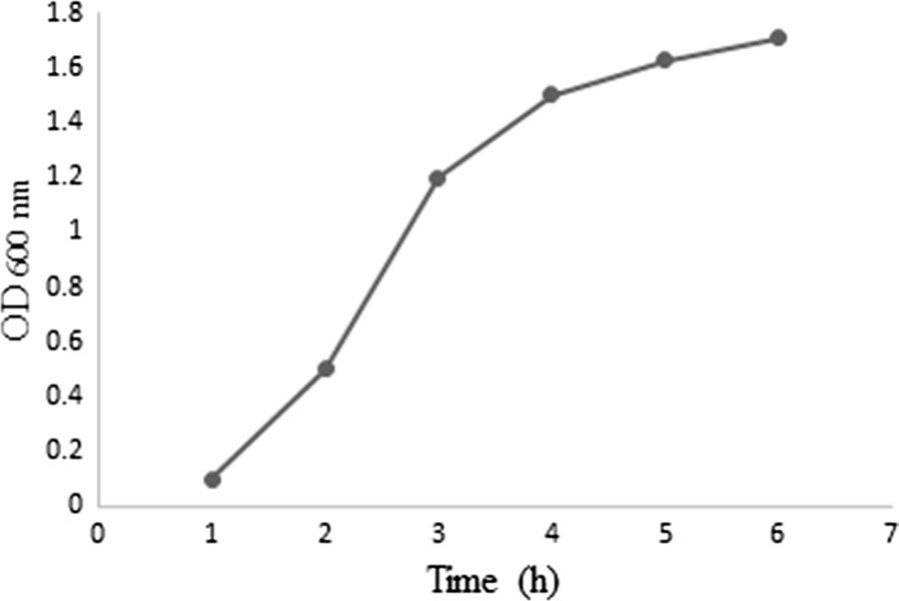
Growth kinetics of *Escherichia coli* in the basal fermentation condition

**Fig. 2:**
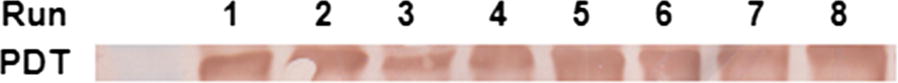
Comparative western blotting of the PDT protein; experimental runs 1–8

The growth curve of *E. coli* BL21 at basal fermentation condition was investigated to monitor the growth pattern (Fig. 1). Comparative western blotting on the experimental runs showed different amount of the PDT protein in each sample that was recognized by the anti-His tag monoclonal antibody (Fig. 2). Figure 2 compares some of the experimental fermentation runs which were employed for the statistical analysis. It was observed that in bacterial growth phase, DO dropped to around 10% after 1 h when the cascade started to compensate for DO drop and purged air or oxygen. The oxygen supply continued afterwards to maintain the DO at desired levels (10%, 30% and 50%).

### Optimization of rPDT production by statistical experimental design

An optimization approach was applied in the present study using the results from a prior screening study. In the previous study, different parameters were screened for effect on rPDT expression in *E. coli* in flask scale. Eight parameters including vector type, bacterial strain, culture medium, expression temperature, shaking speed, IPTG, glucose and antibiotic concentrations were screened using Plackett–Burman statistical design. In the present study, temperature and IPTG concentration as the two most significantly influential factors and dissolved oxygen that could not be precisely controlled in the flask setting, were optimized for the expression of rPDT in pilot scale via fermenter.

### Optimization of the fermentation conditions by Box–Behnken design

To explore the optimum production region of the fusion protein rPDT expression, the main effects of the most significant parameters (X1: T, X_2_: DO, and X_3_: IPTG) and the binary interactions were further investigated. Each independent factor was studied at three levels. Table 2 shows the Box–Behnken design matrix of the coded variables with the experimental results of the rPDT expression. Due to experimental limitations the fermentation runs were performed in one run. However, the center point was performed in five runs and the results showed a satisfactory level of consistence.

**Fig. 3:**
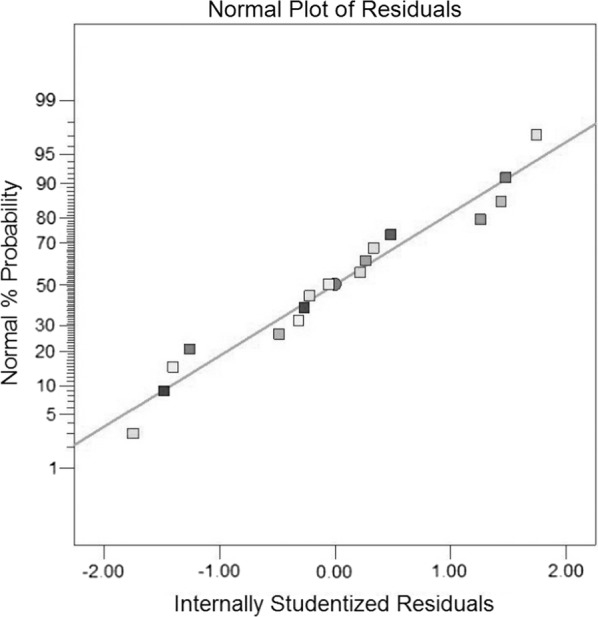
Residual plot, internally Studentized residuals versus predicted values

**Fig. 4:**
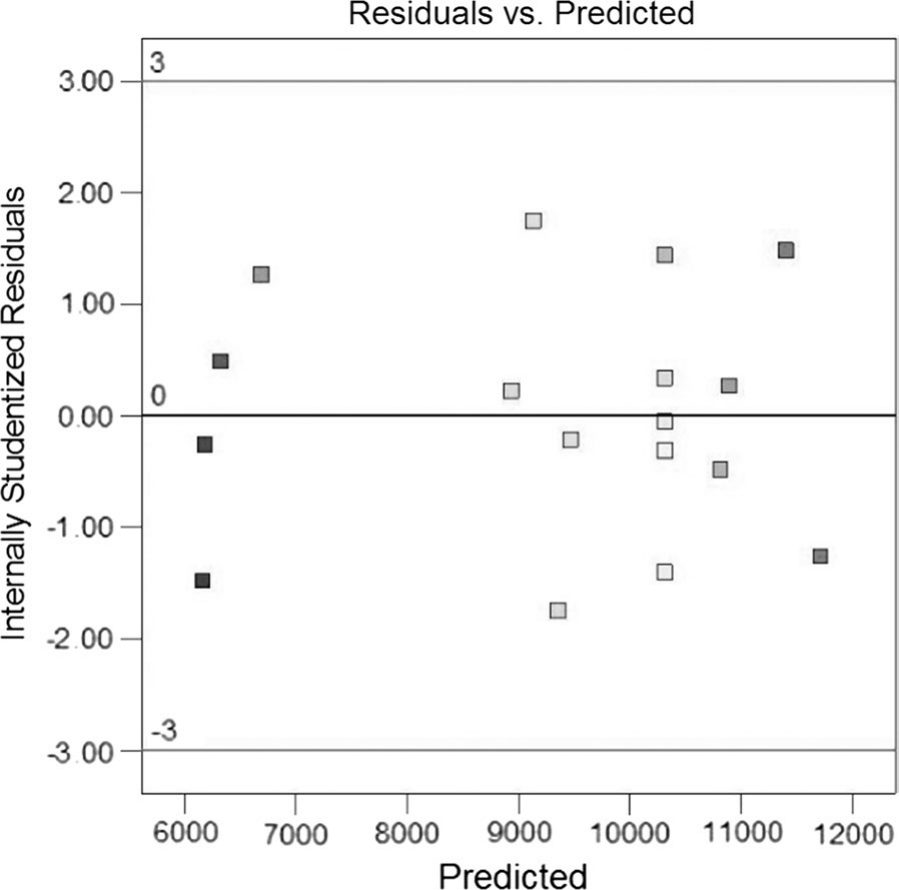
Half-normal probability plot

The plots of normal probability and Studentized residual versus the value predicted by the model reveals no trends that indicates homogeneity of variance in the data and the absence of outlier data in the experimental runs (Figs. 3 and 4).

**Fig. 5:**
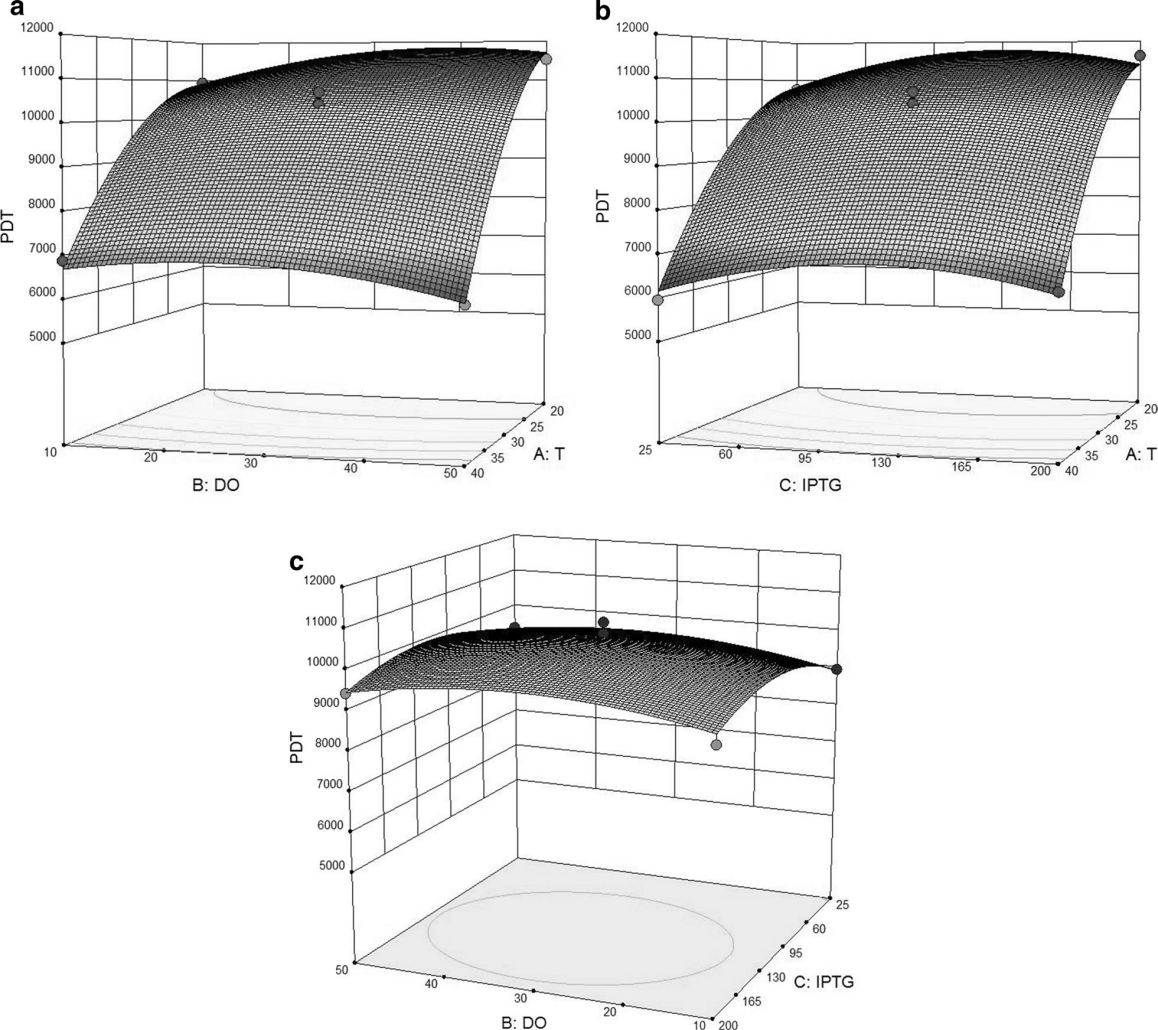
**a** Response surface plot showing the interactive effect of DO and temperature on rPDT expression by *E. coli*. **b** Response surface plot showing the interactive effect of IPTG and temperature on rPDT expression by *E. coli*. **c** Response surface plot showing the interactive effect of DO and IPTG on rPDT expression by *E. coli*

Figure 5 shows the response surface plots for the experimental results. Figure 5a shows that lower levels of temperature favors higher production levels of rPDT. On the other hand, level of the protein remained constant while increasing the dissolved oxygen level in the fermentation medium. Figure 5b also shows the same trend for temperature, however, when the temperature falls down, higher IPTG concentration increases the yield. Figure 5c reveals the relation between DO and IPTG showing that the center point values mostly provide the suitable fermentation conditions. It should also be considered that when low temperature was used for fermentation, higher DO and IPTG would favor the overall yield.

For predicting the optimal response, a second-order polynomial model was fitted onto the experimental data for rPDT expression:

$$\begin{aligned} {\text{Y}} = & \, + 10 3 1 6. 5 5 { } - { 2432}. 9 4 {\text{ X}}_{ 1} + { 77}. 9 5 {\text{ X}}_{ 2} + { 187}. 9 9 {\text{ X}}_{ 3} - { 331}. 1 6 {\text{ X}}_{ 1} {\text{X}}_{ 2} \\ \quad & - 10 7. 1 5 {\text{ X}}_{ 1} {\text{X}}_{ 3} - { 21}. 80{\text{ X}}_{ 2} {\text{X}}_{ 3} - { 996}.0 1 {\text{ X}}_{ 1}^{ 2} - { 448}. 40{\text{ X}}_{ 2}^{ 2} - { 643}.0 9 {\text{ X}}_{ 3}^{ 2} \\ \end{aligned}$$

where X_1_, X_2_, and X_3_ are the culture temperature, DO, and IPTG, respectively. Analysis of variance (ANOVA) showed that the model is significant (F-value = 73.81 and p-value ≤ 0.0001). At the model level, the correlation measures for the estimation of the regression equation are the multiple correlation coefficient R and the determination coefficient R^2^. The closer the value of R is to 1; the better is the correlation between the measured and the predicted values. In this experiment, the value of R was 0.9947 for the production of rPDT. This value indicates a high degree of correlation between the experimental and the predicted values. The value of determination coefficient R^2^ = 0.9896 for rPDT production, being a measure of fit of the model, indicates that about 1.4% of the total variations are not explained by the protein production. In addition, the adjusted R^2^ of 0.9762 and predicted R^2^ of 0.9064 denote that the model explained the data variability with a satisfactory precision (Table 3).

**Table 3:** ANOVA for Box–Behnken design

Source	Sum of squares	df	Mean square	F value	p-value prob > F
Model	5.562E+007	9	6.180E+006	73.81	< 0.0001
A-T	4.735E+007	1	4.735E+007	565.51	< 0.0001
B-DO	48615.39	1	48615.39	0.58	0.4710
C-IPTG	2.827E+005	1	2.827E+005	3.38	0.1088
AB	4.387E+005	1	4.387E+005	5.24	0.0559
AC	45926.63	1	45926.63	0.55	0.4830
BC	1900.39	1	1900.39	0.023	0.8845
A^2^	4.177E+006	1	4.177E+006	49.88	0.0002
B^2^	8.466E+005	1	8.466E+005	10.11	0.0155
C^2^	1.741E+006	1	1.741E+006	20.80	0.0026
Residual	5.862E+005	7	83736.38		
Lack of fit	3.009E+005	3	1.003E+005	1.41	0.3637
Pure error	2.853E+005	4	71316.17		
Cor total	5.621E+007	16	Square		

The optimal levels of the three components as obtained from the maximum point of the polynomial model were estimated using the Design Expert Software, and found to be: temperature 20 °C; DO 30% and IPTG 100 mM. The optimal value of the rPDT production is 1.9 folds in comparison with that of the basal fermentation conditions. This reflects the necessity and the value of optimization process for expression of recombinant proteins.

### Validation of model and growth pattern

The optimized fermentation conditions found in the optimization study were validated experimentally and compared with the calculated data from the model. The model estimated rPDT production was 45 µg/l, while the polynomial model estimated an expression of 43 µg/l (data not shown). The validation study showed a high model accuracy of more than 98%, which is an evidence for the validity of the model in the selected fermentation design space.

## Discussion

The production of recombinant proteins in *E. coli* has been a notable both scientific and industrial issue facing the researchers during recent decades (Ongkudon et al. 2011; Mahalik et al. 2014). In the current study, the production of rPDT was optimized in the pilot fermentation scale while statistical experimental approach was employed. The present study promotes the application of statistical experimental design to elucidate the study design space. Knowing influential parameters and optimizing them can help improve yield and quality attributes of the proteins such as soluble or biologically active products. For example, as Fig. 5 denotes nearly twofold change in protein expression (ranging from about 6000 to 12,000 units) which implies that the DOE model depicts favorable process parameters to optimize the experiments. Three influential parameters including temperature, IPTG concentration and dissolved oxygen were optimized. The application of the fermenter rendered the possibility of controlling propeller speed, dissolved oxygen and gas purge in a cascaded control system that may not be conveniently controlled in flask scale. rPDT protein composed of the immunoprotective S1 fragment of pertussis toxin, the full-length nontoxic diphtheria toxin, and fragment C of tetanus toxin. This fusion protein has been expressed in *E. coli* carrying the recombinant plasmid and has a molecular weight of 161 kDa that is recognizable by specific antibodies. However, rPDT expression, inducible by IPTG, lacks satisfactory yield (Aminian et al. 2007). In this study, the rPDT production was optimized during two phases of screening and optimization studies. The results showed that the protein expression at lower temperature level favored expression yield that could theoretically improve soluble production. The central levels of DO and IPTG served the optimum expression of rPDT protein. Nonetheless, the experimental set-up did not allow the temperature to be controlled at levels lower than 20 °C. In the present study, parameters like pH, antibiotic concentration and culture medium were kept constant. However, the bacterial OD at 600 nm could reach to 1.6 at the maximum as the plateau in the growth phase. It has been shown that the *E. coli* culture density could reach to high ranges which lead to significantly higher overall protein expression (Shiloach and Fass 2005). Temperature has been shown to favor cell growth but during the induction phase disfavors the soluble protein expression and results in inclusion body formation. However, in the present study, low temperature contributed to production yield as the results denote. On the other hand, low temperature could compromise the plasmid stability and the overall production yield of soluble protein (Sørensen and Mortensen 2005). Papaneophytou et al. optimized the post-induction temperature for the expression of RANKL in *E. coli* in five levels (18, 22, 26, 30, and 34 °C). They found out that high temperature of 30 °C or higher in 24 h decreased the yield while culture at low temperature (20 °C) and time of 4 h yielded favored soluble protein production. In addition, soluble protein expression was reached its maximum with both temperature and time to an optimum value then decreased (Papaneophytou et al. 2013). Yari et al. studied the effect of temperature on the expression of the recombinant BoNT/A-Hc in *E. coli*. Temperature (30 and 37 °C) did not have significant effect on the protein expression but low level of temperature favored cell cultivation (Yari et al. 2010, 2012).

Inducer concentration was the other variable that was optimized in this study. Based on the results, the expression increased while IPTG reached its central value. Noteworthy, with an industrial approach the lower the material is consumed the process is more industrially friendly. Papaneophytou et al. studied the effect of IPTG on the expression of RANKL in *E. coli* in five concentrations (0.25, 0.5, 0.75, 1 and 1.25 mM). Protein expression peaked with IPTG concentration to a maximum and then dropped. There was also a significant interaction between time after induction and IPTG. However, IPTG and temperature interaction negatively impacted the protein production (Papaneophytou et al. 2013).

One important issue that the present study tried to shed light on was the influence of dissolved oxygen in the culture medium on the fusion protein production because dissolved oxygen cannot be reliably controlled in flask scale without related O_2_ sensor and Agitation-Gas purge control loop for DO sensor. Some other researchers just tried to control dissolved oxygen level in the *E. coli* culture medium in range including 20–75% (Niccolai et al. 2003; Hajinia et al. 2012; Lee et al. 2002). Although DO had a minor influence on the yield in the current study, but, it can be expected to have significant effects on the yield and the quality of the product in large scale fermentation processes where gradients in parameters like DO exists, also known as dissolved oxygen tension (DOT) (Sandoval-Basurto et al. 2005). As discussed elsewhere, short-term anaerobic condition caused by DOT gradient can compromise the product yield and quality through the diversion of metabolic pathways as directed by induced anaerobic genes (Sandoval-Basurto et al. 2005; Lee 1996; Hannig and Makrides 1998). Inappropriate DO control system or ill-functioning mixing regime of the bioreactor (e.g. baffle design, dead spot, recirculation zone) can undermine adequate DO supply and intensify the DOT gradient (Hambor 2012; Garcia-Ochoa and Gomez 2009).

The quadratic model included two factorial interactions and three factor interactions were excluded from the model. The R^2^ for the model was higher than 0.85. Pillay et al. optimized the baculovirus-insect cell expression system to produce HIV-1 virus-like particles through four parameters including insect cell line, cell density, multiplicity of infection (MOI), and infection time. They found that cell density and infection time significantly affected the expression, but MOI did not (Pillay et al. 2009). Ongkudon and coworkers optimized the expression of plasmid-based measles vaccine (pcDNA3F) harbored in *E. coli* DH5α through medium optimization and pH-temperature induction techniques using RSM. They could increase the expression yield by 1.75 folds (Ongkudon et al. 2011). This research provides a remarkable process optimization sample for pilot scale production of complex recombinant fusion protein vaccines.

## References

[CR1] Aminian M, Sivam S, Lee CW, Halperin SA, Lee SF (2007). Expression and purification of a trivalent pertussis toxin–diphtheria toxin–tetanus toxin fusion protein in *Escherichia coli*. Protein Expr Purif.

[CR2] Andersen DC, Krummen L (2002). Recombinant protein expression for therapeutic applications. Curr Opin Biotechnol.

[CR3] Assenberg R, Wan PT, Geisse S, Mayr LM (2013). Advances in recombinant protein expression for use in pharmaceutical research. Curr Opin Struct Biol.

[CR4] Bayat Y, Zarandi M, Khadiv-Parsi P, Beni AS (2015). Statistical optimization of the preparation of HNIW nanoparticles via oil in water microemulsions. Cent Eur J Energ Mater.

[CR5] Eisel U, Jarausch W, Goretzki K, Henschen A, Engels J, Weller U, Hudel M, Habermann E, Niemann H (1986). Tetanus toxin: primary structure, expression in *E. coli*, and homology with botulinum toxins. EMBO J.

[CR6] Ellis RW (2001). Technologies for the design, discovery, formulation and administration of vaccines. Vaccine.

[CR7] Esposito D, Chatterjee DK (2006). Enhancement of soluble protein expression through the use of fusion tags. Curr Opin Biotechnol.

[CR8] Garcia-Ochoa F, Gomez E (2009). Bioreactor scale-up and oxygen transfer rate in microbial processes: an overview. Biotechnol Adv.

[CR9] Hajinia E, Fatemi SSA, Karkhane AA, Safekordi AA, Yakhchali B (2012). Optimization of secretory expression of recombinant hGM-CSF in high cell density cultivation of recombinant *Escherichia coli* using Taguchi statistical method. IJB.

[CR10] Hambor JE (2012). Bioreactor design and bioprocess controls for industrialized cell processing. BioProcess Int.

[CR11] Hannig G, Makrides SC (1998). Strategies for optimizing heterologous protein expression in *Escherichia coli*. Trends Biotechnol.

[CR12] Hussain MA, Shaik AN, Sechi LA, Ranjan S, Alvi A, Ahmed I, Ranjan A, Mukhopadhyay S, Ahmed N (2008). Isocitrate dehydrogenase of *Helicobacter pylori* potentially induces humoral immune response in subjects with peptic ulcer disease and gastritis. PLoS ONE.

[CR13] Kebriaeezadeh A, Nassiri Koopaei N, Abdollahiasl A, Nikfar S, Mohamadi N (2013). Trend analysis of the pharmaceutical market in Iran; 1997–2010; policy implications for developing countries. DARU.

[CR14] Khoshayand F, Goodarzi S, Shahverdi AR, Khoshayand MR (2011). Optimization of culture conditions for fermentation of soymilk using *Lactobacillus casei* by response surface methodology. Probiotics Antimicrob Proteins.

[CR15] Lee SY (1996). High cell-density culture of *Escherichia coli*. Trends Biotechnol.

[CR16] Lee SF, Halperin SA, Knight JB, Tait A (2002). Purification and immunogenicity of a recombinant *Bordetella pertussis* S1S3FHA fusion protein expressed by *Streptococcus gordonii*. Appl Environ Microbiol.

[CR17] Mahalik S, Sharma AK, Mukherjee KJ (2014). Genome engineering for improved recombinant protein expression in *Escherichia coli*. Microb Cell Fact.

[CR18] Marini G, Luchese MD, Argondizzo APC, de Góes ACMA, Galler R, Alves TLM, Medeiros MA, Larentis AL (2014). Experimental design approach in recombinant protein expression: determining medium composition and induction conditions for expression of pneumolysin from *Streptococcus pneumoniae* in *Escherichia coli* and preliminary purification process. BMC Biotechnol.

[CR19] Niccolai A, Fontani S, Kapat A, Olivieri R (2003). Maximization of recombinant *Helicobacter pylori* neutrophil activating protein production in *Escherichia coli*: improvement of a chemically defined medium using response surface methodology. FEMS Microbiol Lett.

[CR20] Ongkudon CM, Pickering R, Webster D, Danquah MK (2011). Cultivation of *E. coli* carrying a plasmid-based Measles vaccine construct (4.2 kbp pcDNA3F) employing medium optimisation and pH-temperature induction techniques. Microb Cell.

[CR21] Papaneophytou CP, Kontopidis G (2014). Statistical approaches to maximize recombinant protein expression in *Escherichia coli*: a general review. Protein Expr Purif.

[CR22] Papaneophytou CP, Rinotas V, Douni E, Kontopidis G (2013). A statistical approach for optimization of RANKL overexpression in *Escherichia coli*: purification and characterization of the protein. Protein Expr Purif.

[CR23] Pavlou AK, Reichert JM (2004). Recombinant protein therapeutics—success rates, market trends and values to 2010. Nat Biotechnol.

[CR24] Pillay S, Meyers A, Williamson AL, Rybicki EP (2009). Optimization of chimeric HIV-1 virus-like particle production in a baculovirus-insect cell expression system. Biotechnol Prog.

[CR25] Rosano GL, Ceccarelli EA (2014). Recombinant protein expression in *Escherichia coli*: advances and challenges. Front Microbiol.

[CR26] Sandoval-Basurto EA, Gosset G, Bolívar F, Ramírez OT (2005). Culture of *Escherichia coli* under dissolved oxygen gradients simulated in a two-compartment scale-down system: metabolic response and production of recombinant protein. Biotechnol Bioeng.

[CR27] Shiloach J, Fass R (2005). Growing *E. coli* to high cell density—a historical perspective on method development. Biotechnol Adv.

[CR28] Sørensen HP, Mortensen KK (2005). Advanced genetic strategies for recombinant protein expression in *Escherichia coli*. J Biotechnol.

[CR29] Soria-Guerra RE, Alpuche-Solís AG, Rosales-Mendoza S, Moreno-Fierros L, Bendik EM, Martínez-González L, Korban SS (2009). Expression of a multi-epitope DPT fusion protein in transplastomic tobacco plants retains both antigenicity and immunogenicity of all three components of the functional oligomer. Planta.

[CR30] Towbin H, Staehelin T, Gordon J (1979). Electrophoretic transfer of proteins from polyacrylamide gels to nitrocellulose sheets: procedure and some applications. Proc Natl Acad Sci.

[CR31] Yari K, Fatemi SSA, Tavallaei M (2010). Optimization of the BoNT/A-Hc expression in recombinant *Escherichia coli* using the Taguchi statistical method. Biotechnol Appl Biochem.

[CR32] Yari K, Fatemi SSA, Tavallaei M (2012). High level expression of recombinant BoNT/A-Hc by high cell density cultivation of *Escherichia coli*. Bioprocess Biosyst Eng.

